# Comparison of biomechanical effects of polyetheretherketone (PEEK) rods and titanium rods in lumbar long-segment instrumentation: a finite element study

**DOI:** 10.3389/fbioe.2024.1416046

**Published:** 2024-07-11

**Authors:** Chao Li, Yao Zhao, Longtao Qi, Beiyu Xu, Lei Yue, Ranlyu Zhu, Chunde Li

**Affiliations:** Department of Orthopedics, Peking University First Hospital, Beijing, China

**Keywords:** lumbar degenerative disease, posterior lumbar instrumentation, PEEK, dynamic stabilization, adjacent segment degeneration, finite element analysis

## Abstract

**Introduction:**

Polyetheretherketone (PEEK) lumbar fusion rods have been successfully used in short-segment posterior instrumentation to prevent adjacent segment degeneration. However, limited studies have reported their application in lumbar long-segment instrumentation. This study aimed to compare the biomechanical performances of PEEK rods and titanium rods in lumbar long-segment instrumentation using finite element (FE) models, with the expectation of providing clinical guidance.

**Methods:**

A lumbar FE model (A) and four lumbar fixation FE models (BI, CI, BII, CII) of the L1–S1 vertebral body were developed using CT image segmentation (A: intact model; BI: intact model with L2–S1 PEEK rod internal fixation; CI: intact model with L2–S1 titanium rod internal fixation; BII: intact model with L3–S1 PEEK rod internal fixation; CII: intact model with L3–S1 titanium rod internal fixation). A 150-N preload was applied to the top surface of L1, similar to the intact model. The stresses on the lumbar intervertebral disc, facet joint, pedicle screws, and rods were calculated to evaluate the biomechanical effect of the different fixation procedures in lumbar long-segment instrumented surgery.

**Results:**

Under the four physiological motion states, the average stresses on the adjacent segment intervertebral disc and facet joint in all fixation models were greater than those in the intact model. Furthermore, the average stresses on the adjacent segment intervertebral disc and facet joint were greater in models CI and CII than in models BI and BII, respectively. The average stresses on the pedicle screws and rods were decreased in models BI and BII compared with models CI and CII under the four physiological motion states, respectively.

**Discussion:**

The PEEK rod internal fixation system may have better biomechanical properties than the titanium rod internal fixation system in delaying adjacent segment degeneration, improving the lumbar function of postoperative patients, and reducing the risk of screw loosening and breakage in lumbar long-segment instrumentation.

## 1 Introduction

Posterior lumbar fusion and internal fixation is a classic surgical method for treatment of lumbar degenerative diseases (Li et al., 2013; Du et al., 2020; Pei et al., 2023; Zhang et al., 2023). Titanium alloy rods have been widely used in lumbar fusion internal fixation systems because of their close biomechanical properties to bone, excellent biocompatibility, and provision of sufficient strength under physiological loads (Kurtz and Devine, 2007; Wedemeyer et al., 2007; Ponnappan et al., 2009). However, the titanium alloys used in screw-rod fixation systems have a much higher elastic modulus than bone tissue (110 GPa vs. 0.1–20 GPa), and this can lead to stress concentration in posterior lumbar screw-rod fixation systems and stress shielding of intervertebral bone grafts, thereby increasing the risk of long-term complications such as pseudarthrosis, adjacent segment degeneration (ASD), and screw loosening or breakage (De Iure et al., 2012; Wu et al., 2020; Fan et al., 2021).

In recent years, the latest advancements in posterior spinal fixation have focused on the concept of dynamic stabilization (Khoueir et al., 2007; Greiner-Perth et al., 2016). Dynamic stabilization, which is often used as a non-fusion fixation alternative, is theoretically superior to traditional stiff fixation and can minimize ASD. When applied to fusion methods, enhanced dynamic stabilization provides additional load sharing to the anterior column and reduces stress at the bone-screw interface. However, decreased stability of internal fixation is an inherent risk factor for pseudarthrosis. The ideal fixation system would maximize the fusion rate by providing sufficient stability without excessive rigidity to minimize stress on the adjacent segment.

Polyetheretherketone (PEEK) is a fully biocompatible biomaterial that is increasingly being used for spinal implants. Its elastic modulus (3.6 GPa) is much lower than that of titanium alloys and closer to that of bone, and thus PEEK can better balance the load distribution between the anterior and posterior columns (Kurtz and Devine, 2007; Ponnappan et al., 2009; Ma and Tang, 2014; Li et al., 2018; Laubach et al., 2022; Wu et al., 2023). In previous studies, PEEK rods were successfully used for short-segment lumbar internal fixation with good clinical outcomes (Zhao et al., 2022; Li et al., 2023a). However, limited studies have reported their application in long-segment lumbar internal fixation. The present study aimed to compare the biomechanical performances of PEEK rods and titanium rods in lumbar long-segment instrumentation surgery using finite element (FE) models, with the expectation of providing clinical guidance.

## 2 Materials and methods

### 2.1 Development of an FE model of the intact lumbar-sacral spine

**FIGURE 1:**
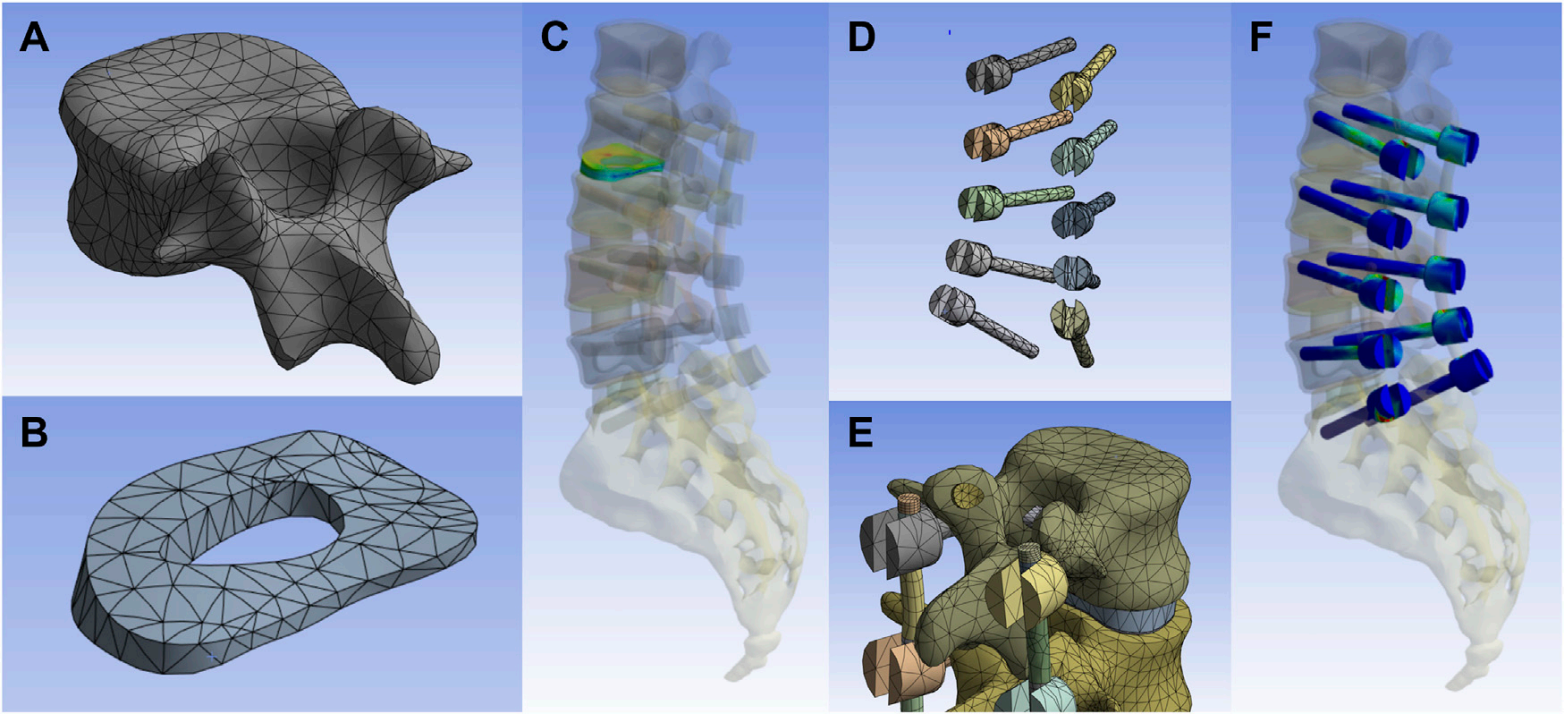
Development of an FE model. **(A)** The vertebral body after dividing the grid. **(B,C)** The Annulus fibrosus and its combination relationship in the whole model. **(D)** Model of pedicle screw. **(E)** Assembly relationship of pedicle screw, rods, facet joints and vertebrae. **(F)** Assembly relationship of pedicle screws in the overall model.

To establish an FE model of the intact lumbar-sacral spine, 0.5-mm thick CT images of the L1–S1 vertebral body in a healthy male subject were obtained from Peking University First Hospital. Mimics 11 (Materialise, Belgium), a medical image processing software program, was used to reconstruct the surface geometries of the vertebrae and sacrum. The models were then exported to Geomagics Studio 13.0 (Raindrop Geomagics, United States) and converted to WRP files. The separate vertebrae and sacrum models were integrated with origin alignment in SolidWorks 2020 (Dassault Systems, France) and the intervertebral discs and posterior elements were created based on the reconstructed vertebrae. The thicknesses of the cortical bones and endplates were set at 2 mm and 0.5 mm, respectively. The material properties of the FE model were derived from previous studies (Ambati et al., 2015; Zhang et al., 2022; Li et al., 2023b) and are detailed in Table 1. The final FE model of the lumbar-sacral spine was composed of L1–L5, sacrum, coccyx, and five intervertebral discs (Figure 1).

**TABLE 1:** Material properties of finite element models.

Material	Young’s modulus (MPa)	Poisson’s ratio
Bony Structure		
Cancellous bone	100	0.2
Cortical bone	12,000	0.3
Posterior elements	3,500	0.25
Endplates	3,000	0.25
Sacrum and coccyx	3,500	0.25
Intervertebral Disc		
Nuclei pulposi	1	0.49
Annuli fibrosi	4.2	0.45
Implants		
Pedicle screw (Titanium)	110,000	0.28
Rod (PEEK)	3,600	0.25
Rod (Titanium)	110,000	0.28

### 2.2 Development of FE models of the implanted lumbar-sacral spine

To simulate implantation and fixation, four FE models were established by modifying the intact model based on posterior spinal surgery. Briefly, we modeled implantation of internal fixation at L2–S1 and L3–S1 using rod fixation systems over the length of five segments and four segments, respectively. We then assigned the materials in each section as whole PEEK or whole titanium, generating the four implanted FE models. The titanium pedicle screws had a diameter of 6.5 mm. The PEEK rods and titanium rods had a diameter of 5.5 mm. All of the implants were meshed as three-dimensional solid elements.

**FIGURE 2:**
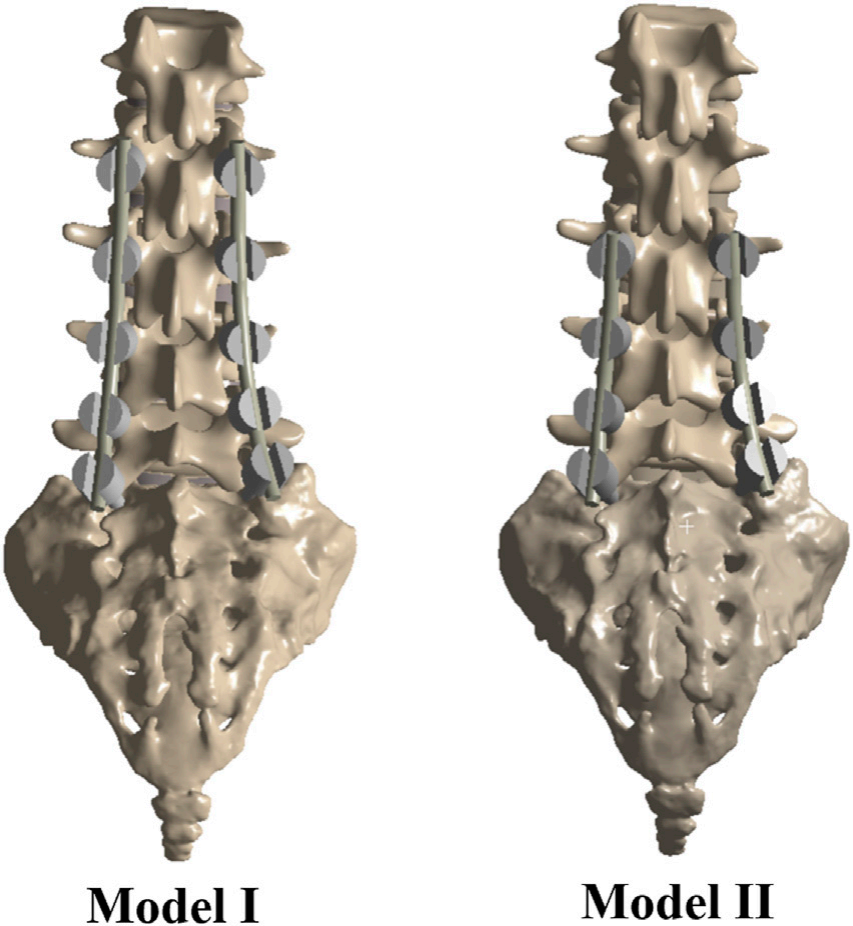
The FE models of the implanted lumbar-sacral spine constructed for the present study. Model I: intact model with L2–S1 internal fixation; Model II: intact model with L3–S1 internal fixation.

As shown in Figure 2, the FE models of the implanted lumbar-sacral spine were established for the study. Model A was the intact model, model BI was the intact model with L2–S1 PEEK rod internal fixation, model CI was the intact model with L2–S1 titanium rod internal fixation, model BII was the intact model with L3–S1 PEEK rod internal fixation, and model CII intact model with L3–S1 titanium rod internal fixation. The surgical operation was simulated in the models with internal fixation, and comparative analysis of the mechanical properties was performed. The models provided a realistic reproduction of the surgical operation and enabled analysis of the mechanical properties.

### 2.3 FE analysis of the implanted lumbar-sacral spine

The interactions between the pedicle screws and vertebrae and between the pedicle screws and rods were defined as bonded. The facet joints were assigned no separation with two adjacent vertebrae. All faces of the sacrum and coccyx were fixed in all directions. A 150-N preload and a 10-Nm moment were applied to the top surface of L1 to validate the loading conditions in the intact model, based on a previous *in vitro* study (Yamamoto et al., 1989). For the implanted models, a displacement-controlled FE analysis was used and the loads were applied in two steps. First, a 150-N preload was applied to the top surface of L1, similar to the intact model. Second, to determine the equivalent moment, an iterative process of moment increasing from 10 Nm was used and the approximated moment was applied to the top surface of L1, reaching the same range of motion (ROM) as the intact model. The accuracy of the ROM was 0.01 degrees. The two-step load procedure was based on several works in accordance with a hybrid testing protocol (Zhong et al., 2009; Zhang et al., 2022).

### 2.4 Data analyses

The stresses on the lumbar intervertebral disc and facet joint were used to evaluate the biomechanical effects on these areas after the different internal fixation procedures (titanium or PEEK rods) under the conditions of flexion, extension, lateral bending, and axial rotation, which are related to the risk of ASD. The stresses on the screws and rods were calculated to evaluate the risk of instrument failure.

## 3 Results

In this study, an L1–S1 vertebral body model was reconstructed and implanted with internal fixation systems for FE analysis. The biomechanical properties under four physiological motion states were evaluated.

### 3.1 Verification of model validity

**FIGURE 3:**
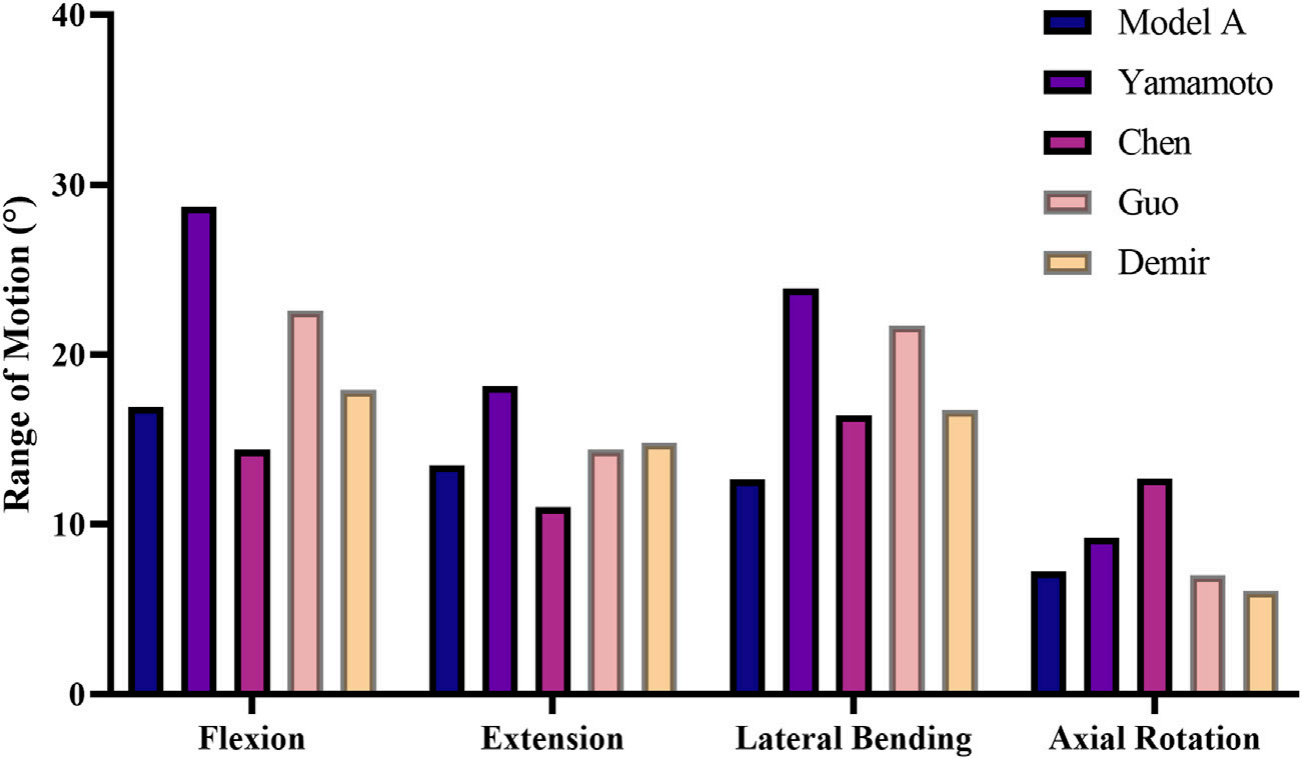
Comparison of the ROM in intact model A with other ROMs in previous studies.

To validate the L1–S1 intact lumbar spine FE model, the ROM was calculated for model A and compared with the ROMs in previous *in vitro* studies and FE studies under the same loads (Yamamoto et al., 1989; Chen et al., 2001; Guo and Yin, 2019; Demir et al., 2020). As shown in Figure 3, excellent agreement was noted between the experimental results and the calculated results. The ROM in model A was concluded to be in good agreement with the results of the other studies, thus confirming the validity of the experimental FE model.

### 3.2 Stresses on the intervertebral discs

**FIGURE 4:**
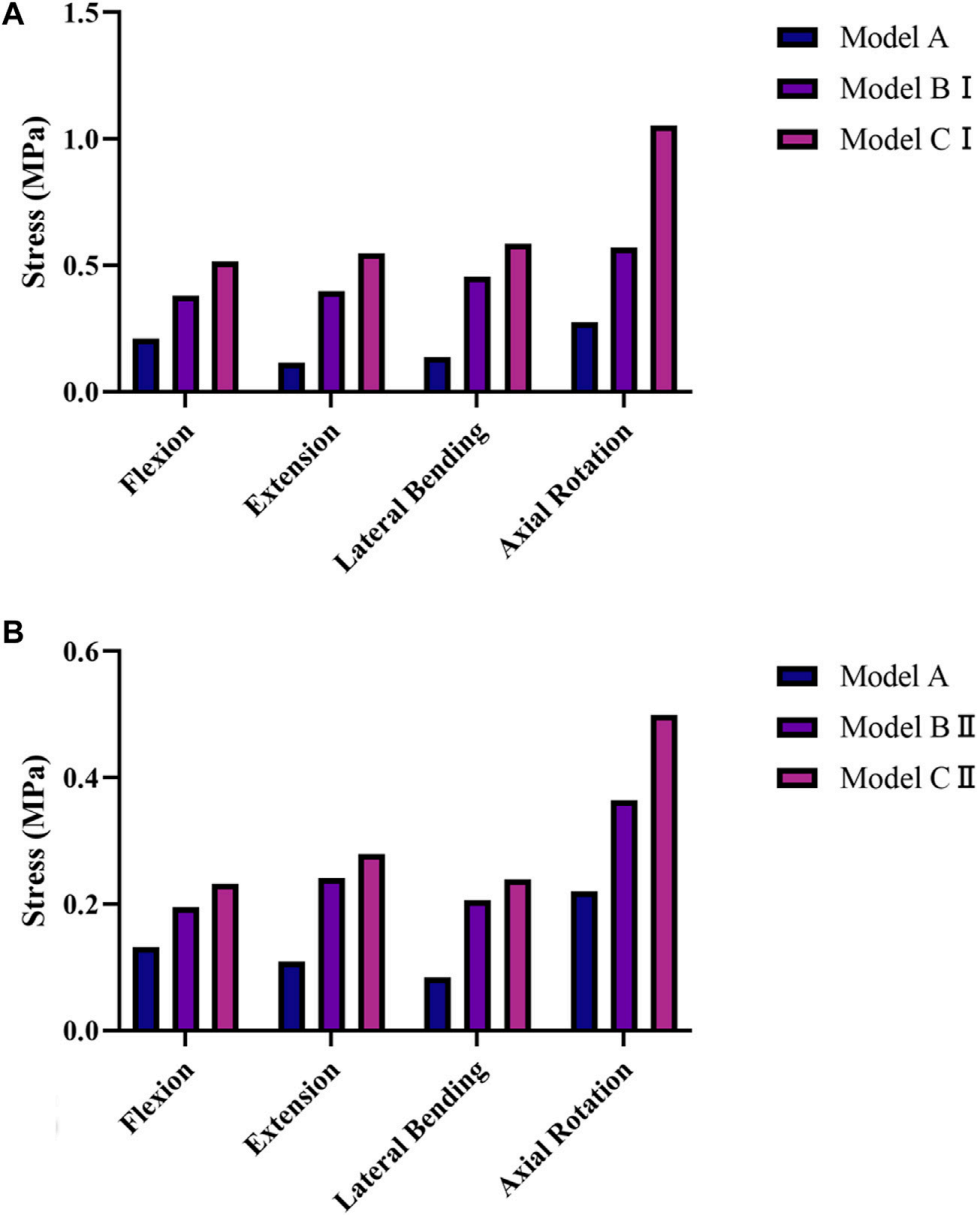
Average von Mises stresses on the intervertebral discs in the five models under the four physiological motion states. **(A)** Average von Mises stresses on the L1–L2 intervertebral disc in models A, BI, and CI under the four physiological motion states. **(B)** Average von Mises stresses on the L2–L3 intervertebral disc in models A, BII, and CII under the four physiological motion states.

The average stresses on the L1–L2 and L2–L3 intervertebral discs in the five models are shown in Figure 4. Compared with model A, the average stresses on the L1–L2 intervertebral disc in model BI and model CI and the average stresses on the L2–L3 intervertebral disc in model BII and model CII were increased under the four physiological motion states. In addition, the average stress on the L1–L2 intervertebral disc in model CI was greater than that in model BI under the four physiological motion states; similarly, the average stress on the L2–L3 intervertebral disc in model CII was greater than that in model BII under the four physiological motion states. The average stress on the L1–L2 intervertebral disc was increased by an average of 167.0% for model BI and 283.8% for model CI compared with model A under the four physiological motion states. The average stress on the L2–L3 intervertebral disc was increased by an average of 95.1% for model BII and 135.6% for model CII compared with model A under the four physiological motion states. These findings may be used to solve the problem of disc degeneration on the adjacent segment in patients after lumbar long-segment instrumented surgery.

### 3.3 Stresses on the facet joints

**FIGURE 5:**
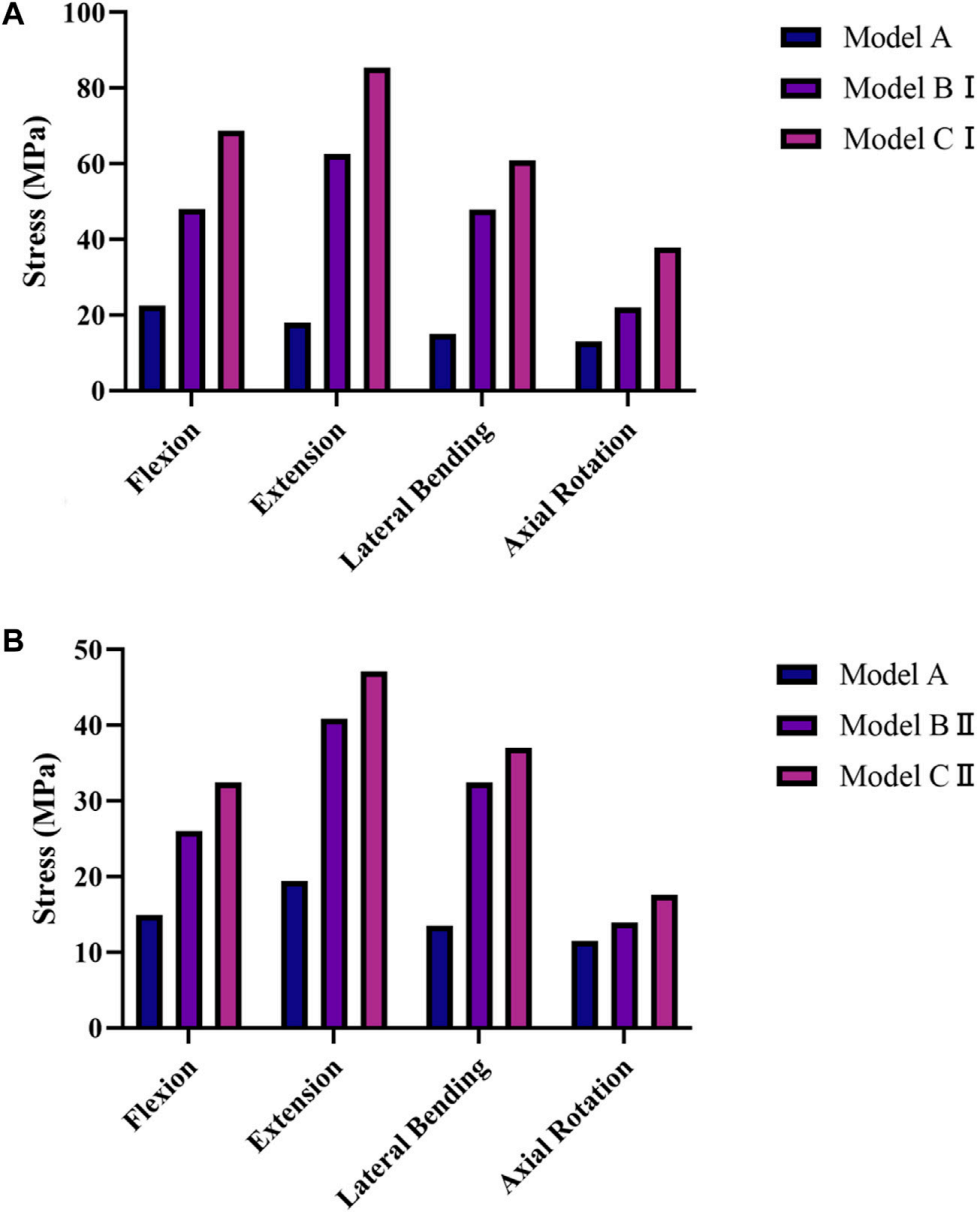
Average von Mises stresses on the facet joints in the five models under the four physiological motion states. **(A)** Average von Mises stresses on the L1–L2 facet joint in models A, BI, and CI under the four physiological motion states. **(B)** Average von Mises stresses on the L2–L3 facet joint in models A, BII, and CII under the four physiological motion states.

The average stresses on the L1–L2 and L2–L3 facet joints in the five models are shown in Figure 5. Compared with model A, the average stresses on the L1–L2 facet joint in model BI and model CI and the L2–L3 facet joint in model BII and model CII were increased under the four physiological motion states. In addition, the average stress on the L1–L2 facet joint in model CI was greater than that in model BI under the four physiological motion states; similarly, the average stress on the L2–L3 facet joint in model CII was greater than that in model BII under the four physiological motion states. The average stress on the L1–L2 facet joint was increased by an average of 162.8% for model BI and 269.5% for model CI compared with model A under the four physiological motion states. The average stress on the L2–L3 facet joint was increased by an average of 86.7% for model BII and 121.9% for model CII compared with model A under the four physiological motion states. These findings may be used to solve the problem of facet joint degeneration on the adjacent segment in patients after lumbar long-segment instrumented surgery.

### 3.4 Stresses on the screws and rods

**FIGURE 6:**
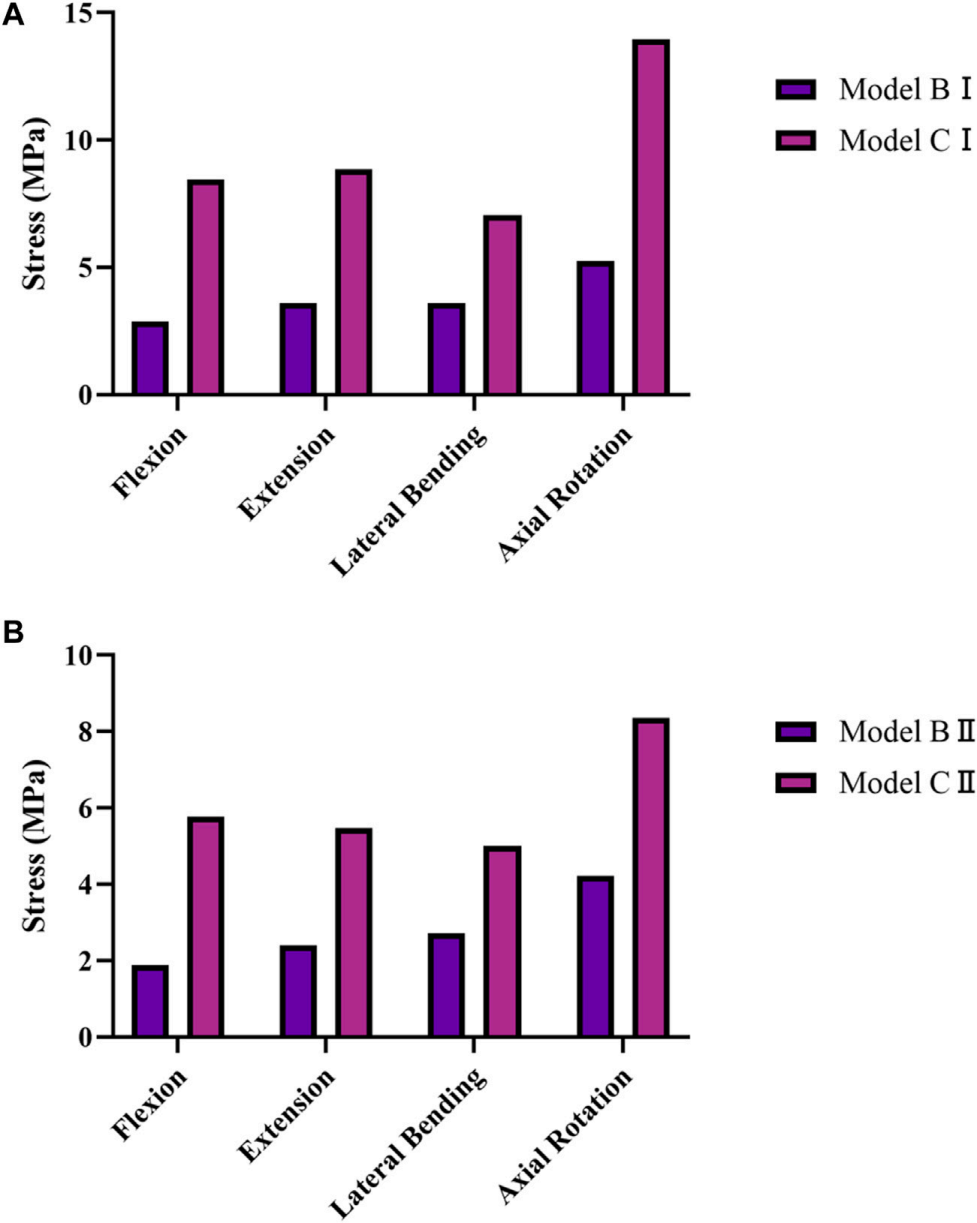
Average von Mises Stress on the pedicle screws in models BI, BII, CI, and CII under the four physiological motion states. **(A)** Average von Mises stresses on the pedicle screws in models BI and CI under the four physiological motion states. **(B)** Average von Mises stresses on the pedicle screws in models BII and CII under the four physiological motion states.

**FIGURE 7:**
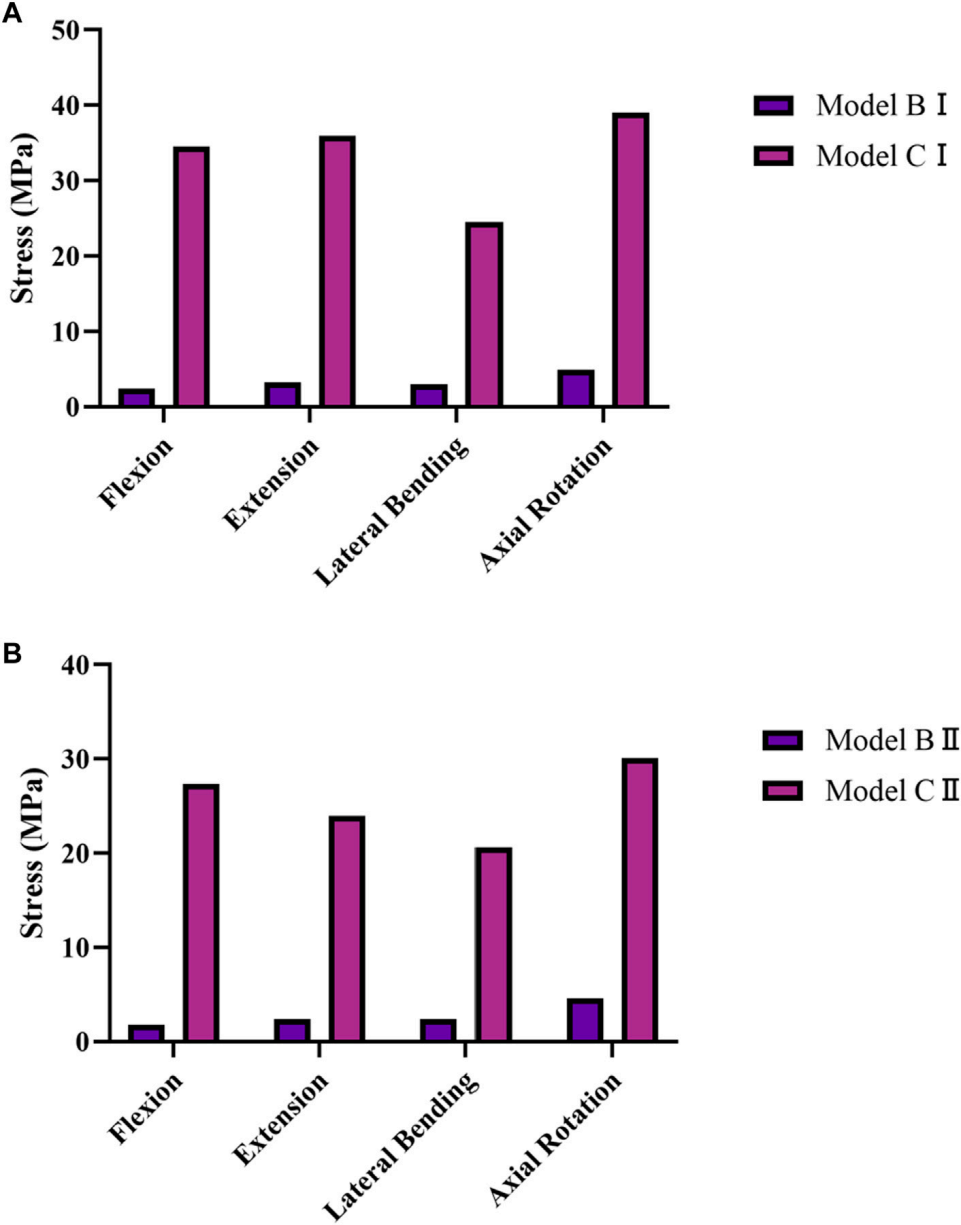
Average von Mises stresses on the rods in models BI, BII, CI, and CII under the four physiological motion states. **(A)** Average von Mises stresses on the rods in models BI and CI under the four physiological motion states. **(B)** Average von Mises stresses on the rods in models BII and CII under the four physiological motion states.

**FIGURE 8:**
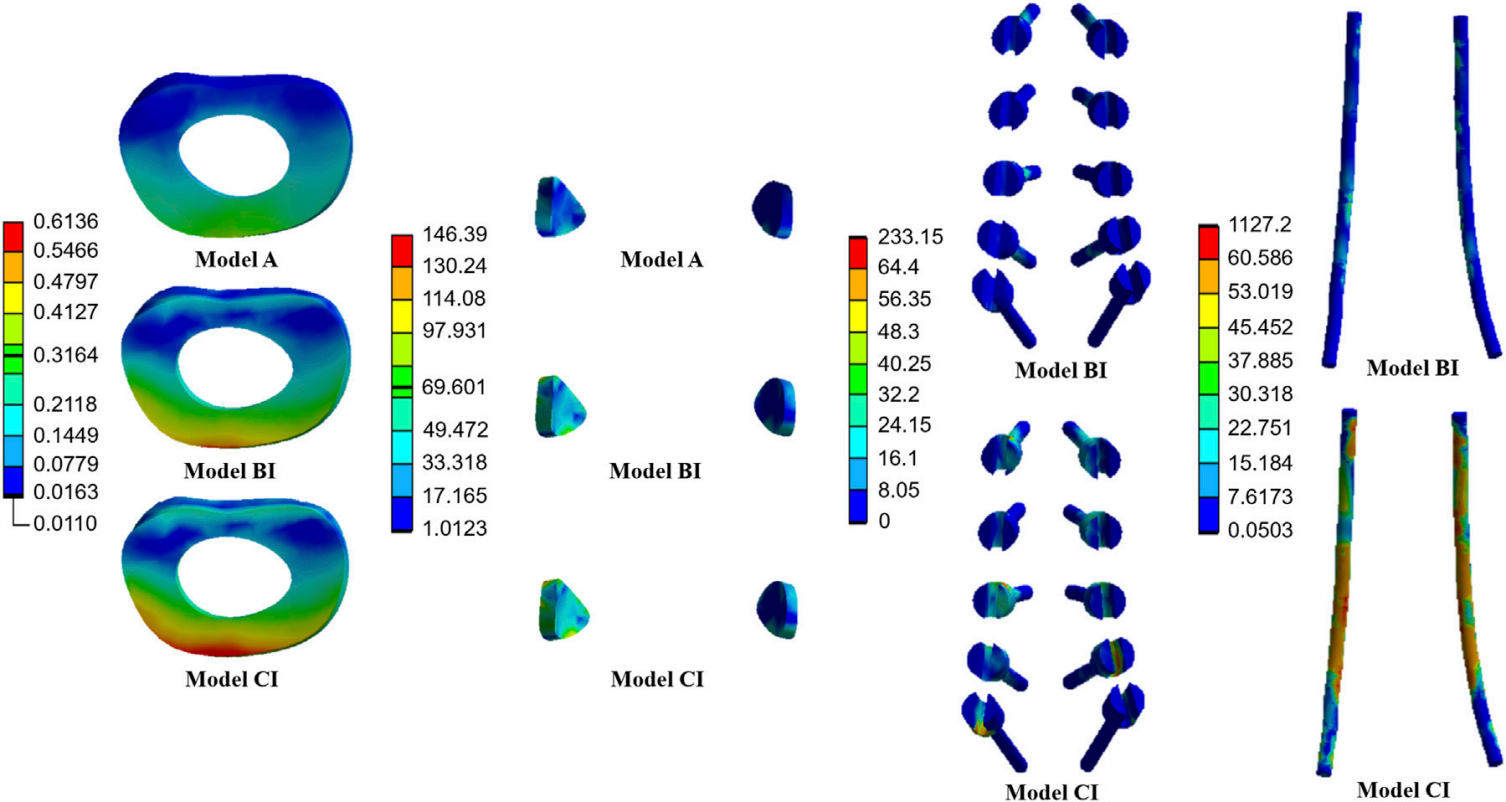
Nephogram of von Mises stress (MPa) on the L1/2 intervertebral discs, L1/2 facet joints, pedicle screws, and rods in flexion condition on Models A, BI, and CI.

**FIGURE 9:**
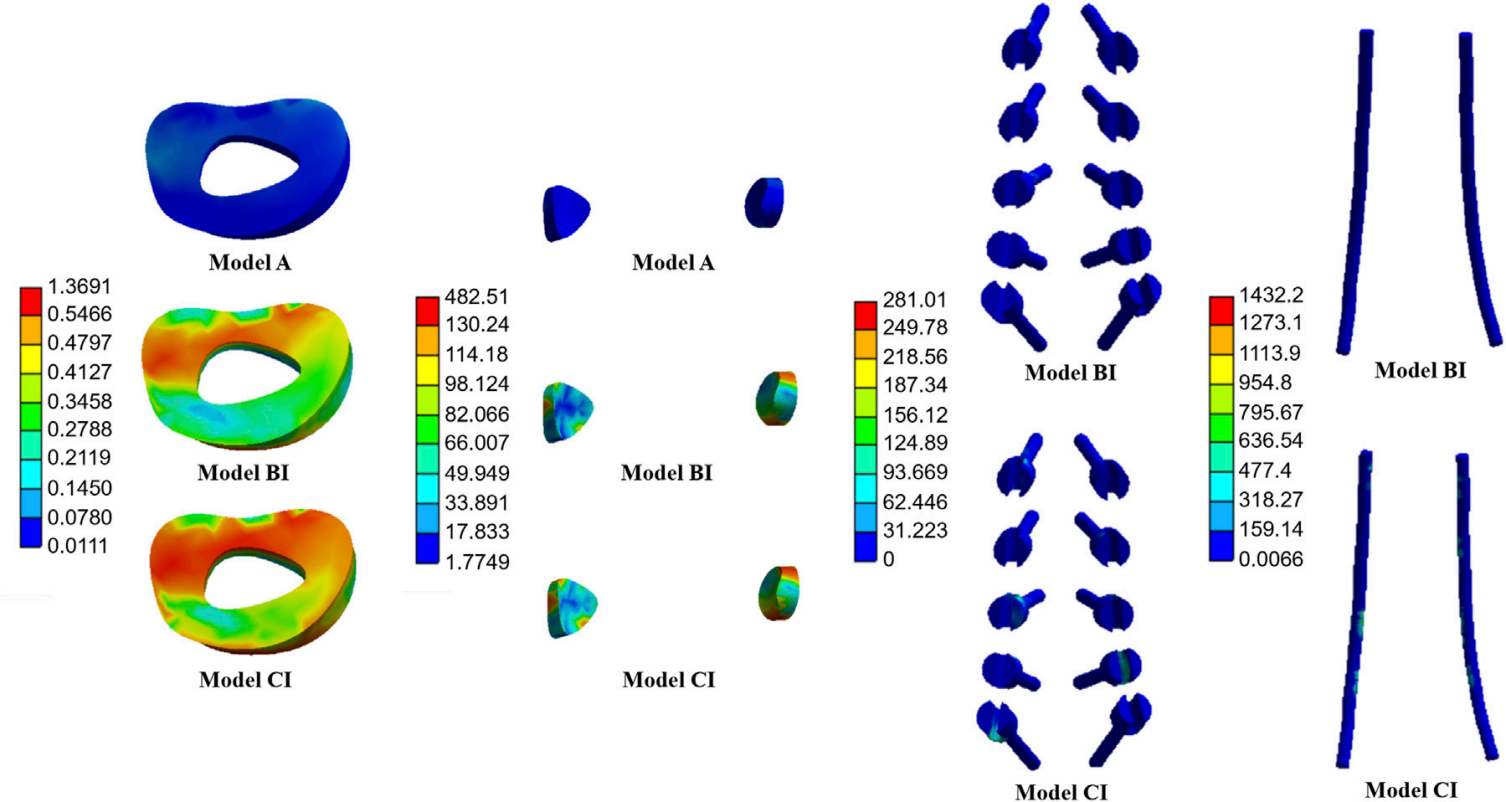
Nephogram of von Mises stress (MPa) on the L1/2 intervertebral discs, L1/2 facet joints, pedicle screws, and rods in extension condition on Models A, BI, and CI.

**FIGURE 10:**
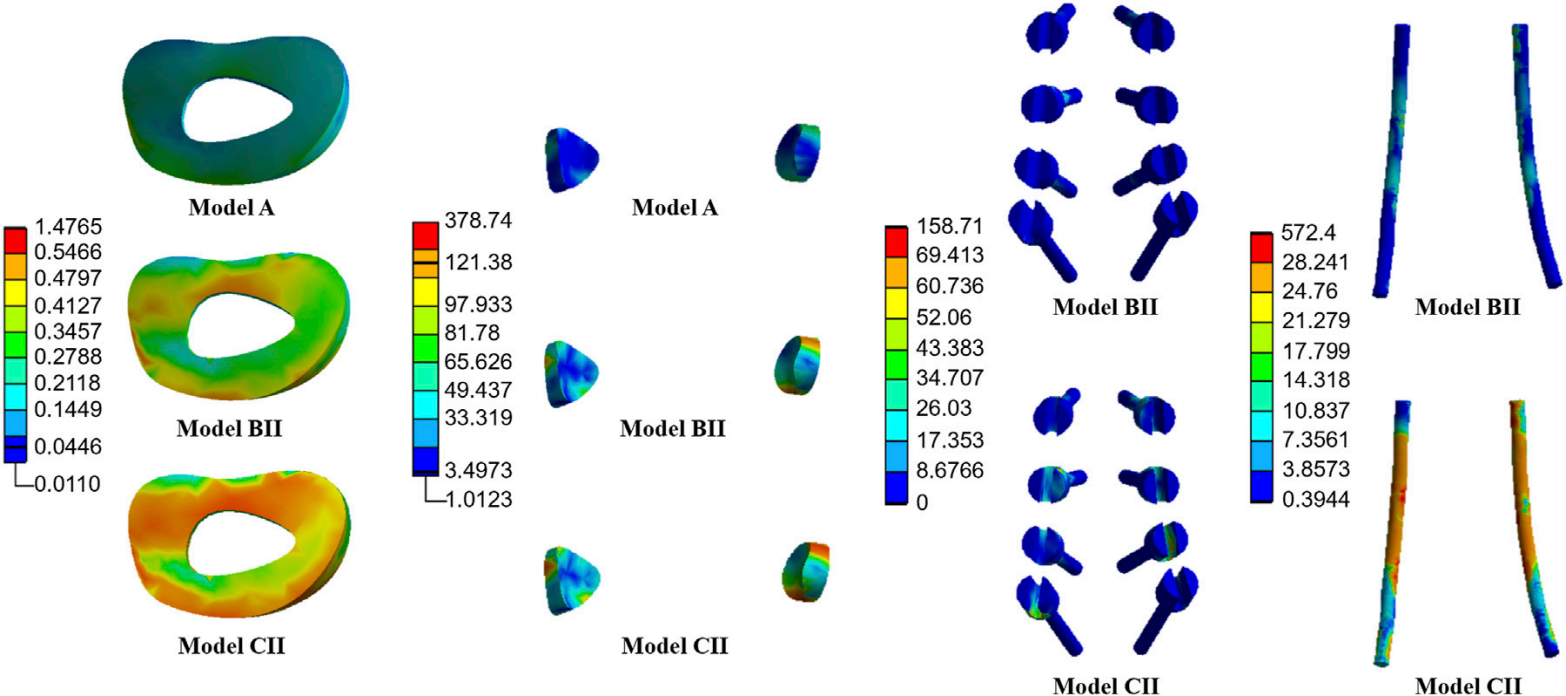
Nephogram of von Mises stress (MPa) on the L2/3 intervertebral discs, L2/3 facet joints, pedicle screws, and rods in flexion condition on Models A, BII, and CII.

**FIGURE 11:**
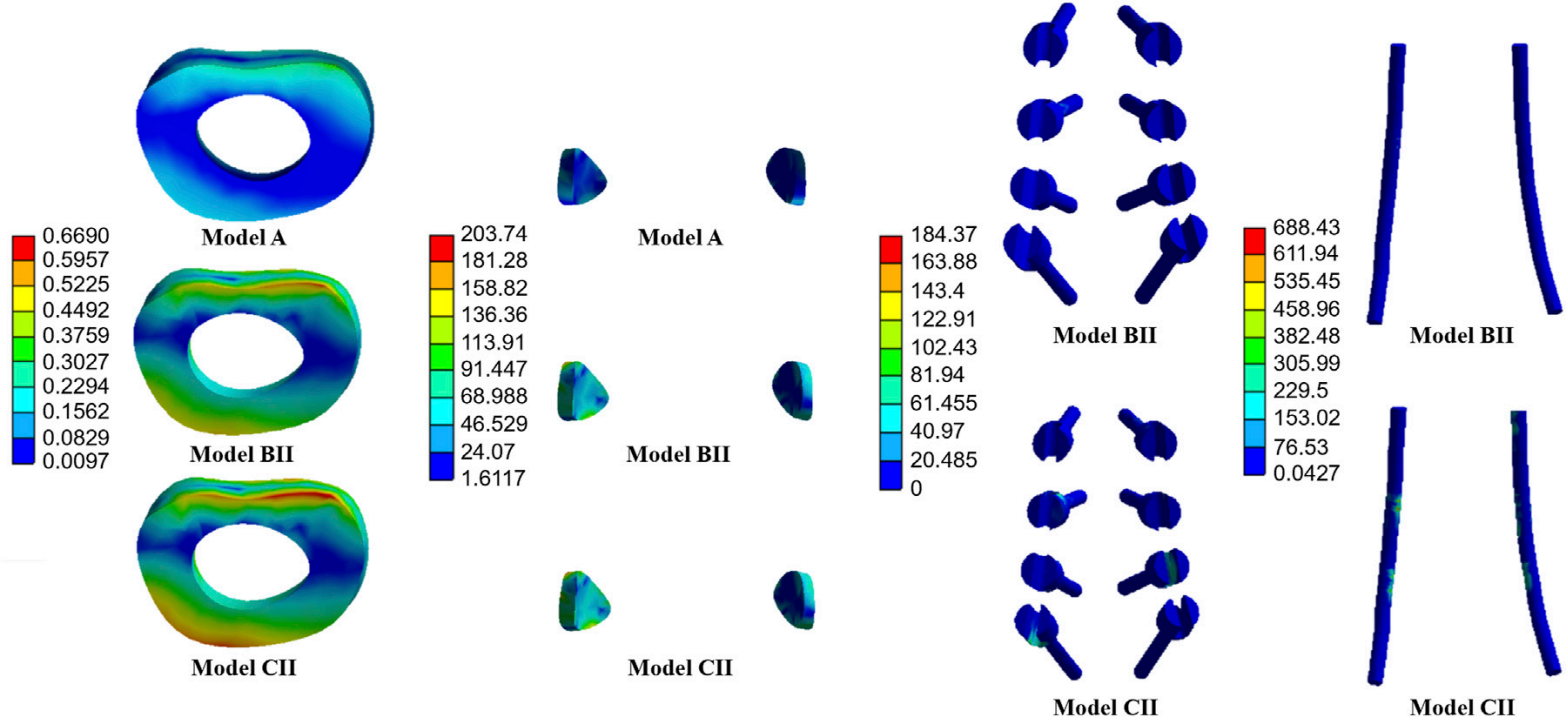
Nephogram of von Mises stress (MPa) on the L2/3 intervertebral discs, L2/3 facet joints, pedicle screws, and rods in extension condition on Models A, BII, and CII.

The average stresses on the screws and rods in the four implanted models with the internal fixation systems are shown in Figures 6, 7, respectively. The average stresses on the screws and rods in models CI and CII were greater than those in models BI and BII under the four physiological motion states, respectively. Compared with model BI, the average stresses on the screws and rods in model CI were increased by an average of 150.0% and 940.8% under the four physiological motion states, respectively. Compared with model BII, the average stresses on the screws and rods in model CII were increased by an average of 129.3% and 903.6% under the four physiological motion states, respectively. These findings may be used to solve the problem of internal fixation system failure and screw-rod fracture in patients after lumbar long-segment instrumented surgery. Additionally, the comparison of the nephogram of von Mises stress on the intervertebral discs, facet joints, pedicle screws, and rods in flexion and extension condition on different models are shown in Figures 8–11, respectively.

## 4 Discussion

Currently, titanium pedicle screw rods (rigid fixation) and PEEK cages have been widely used in posterior lumbar fusion surgery to stabilize the surgical segment and restore the spinal column sequence (Li et al., 2020; Laubach et al., 2022). However, the complications caused by rigid fixation, such as ASD, pseudarthrosis, screw loosening, loss of motion, and back pain, significantly reduce the quality of life of patients, and thus the concept of semi-rigid fixation has been applied in recent years (Khoueir et al., 2007; Greiner-Perth et al., 2016; Nikkhoo et al., 2021). The core point for semi-rigid fixation is that the low-strength material still limits the ROM of the fixed segment, but tends to also reduce the overall structural stiffness of the system, leading to more even distribution of the spinal load between the anterior and posterior columns and preventing various complications caused by the unbalanced load distribution associated with rigid fixation. A less rigid stabilization system can also theoretically preserve part of the rotational motion at the instrumented level and unload any extra stress exposure on adjacent levels (Maragkos et al., 2020; Nikkhoo et al., 2021).

As a representative component for semi-rigid fixation systems, PEEK rods have been highly anticipated by scholars and gradually introduced into posterior spinal fusion since 2007 (Huang et al., 2016). Although previous studies showed that PEEK rods had better load distribution performance than titanium rods and were successfully used for short-segment lumbar internal fixation with good clinical outcomes, doctors remain very cautious about their clinical application in long-segment lumbar internal fixation. One important reason for this is that the biomechanical performance of PEEK rods has not been comprehensively evaluated in long-segment lumbar internal fixation. Moreover, it is difficult to evaluate the biomechanical effects of lumbar fixation in clinical studies. Therefore, the present study was conducted to compare the biomechanical performances of PEEK rods and titanium rods in lumbar long-segment instrumentation surgery using FE models, with the expectation of providing clinical guidance. As the spinal fixation construct is the most essential component of lumbar fusion surgery, this study aimed to investigate the fixation itself. Thus, posterolateral fixation was utilized when simulating the postoperative models with titanium rods and PEEK rods, and bone graft fusion between the transverse processes was neglected, as a common simplification in the literature (Jahng et al., 2013; Jin et al., 2013).

ASD is a common complication after lumbar fusion surgery, because the decrease in ROM of the fixed segment requires compensation by the adjacent segment, and the internal fixation changes the normal mechanical transmission process in the spine. In a previous FE study, Jin et al. (Jin et al., 2013) demonstrated that PEEK rods significantly decreased the stresses on the intervertebral disc and facet joint in the upper adjacent segment compared with titanium rod internal fixation in short-segment lumbar internal fixation surgery. In the present study, the average stresses on the intervertebral disc and facet joint in the upper adjacent segment in all internal fixation models were greater than those in the intact model under all four physiological motion states. Meanwhile, the average stresses on the intervertebral disc and facet joint on the upper adjacent segment in the titanium rod models (CI and CII) were greater than those in the PEEK rod models (BI and BII) under the four physiological motion states, respectively, showing that the use of PEEK rods in lumbar long-segment instrumentation surgery confers the advantage of reduced ASD. However, it is not sufficient to solely evaluate ASD based on the stresses on the intervertebral disc and facet joint in lumbar long-segment instrumentation surgery, and other factors such as intervertebral disc height and sagittal balance should also be taken into account.

The stability of pedicle screw rod internal fixation systems has always been a concern of spine surgeons. In the present study, use of PEEK rods led to lower screw stress than use of titanium rods, leading to a lower risk of screw breakage with PEEK rods in lumbar long-segment instrumentation surgery. This effect may also be attributed to the reduced stress shielding with PEEK rods, which would reduce the load through the posterior hardware. A related study found that PEEK rods have the potential biomechanical advantages of better anterior column load sharing and reduced stress at the bone-to-screw interface (Ponnappan et al., 2009), consistent with the findings in the present study.

A recent study tested the biomechanical properties of PEEK rods and titanium rods *in vitro* using human lumbar spine specimens, and the results indicated that there were no significant differences in the stability provided by PEEK rods and titanium rods under any of the loading modes examined (Yeager et al., 2015). In addition to limiting abnormal segmental motion, maintenance of good anterior and posterior column load distribution in the spine is essential to alleviate various complications. While titanium rods are uniquely suited to provide postoperative stability, they also dramatically alter the physiological loading characteristics of the spine (Ponnappan et al., 2009; Yeager et al., 2015). A titanium pedicle screw rod system was shown to transmit approximately 67% of the axial compressive load during posterior lumbar internal fixation surgery, whereas the posterior column in the natural upright position carries only approximately 20% of the load (Cunningham and Polly, 2002; Ahn et al., 2008). An FE study revealed that PEEK rods carry at least 6% less load than titanium rods (Gornet et al., 2011). In the present study, the results demonstrated the average stresses on the rods were increased in the titanium rod models (CI and CII) under the four physiological motion states compared with the PEEK rod models (BI and BII), respectively. It is speculated that the advantage of the load distribution performance of PEEK rods is mainly due to the reduction in stress concentration in the posterior pedicle screw rod system. However, the stress data indicated that although the stresses on PEEK rods were lower than those on titanium rods, the yield stress was significantly lower on PEEK rods (100 MPa) than on titanium rods (750 MPa), resulting a higher ratio of stress on the rod to yield stress on the rod material for the PEEK rods. Consequently, the PEEK rod system may face a higher risk of rod breakage. In summary, just as there are two sides to a coin, PEEK rods have both advantages and disadvantages. Therefore, further studies are warranted to validate the clinical outcomes for use of PEEK rods in lumbar long-segment instrumentation surgery in clinical practice.

The present experimental study inevitably has some limitations. First, the geometry of the human lumbar spine varies from individual to individual, but the FE model built in this study was based on a single patient. Therefore, additional samples are needed for future validation. Second, the theoretical numerical model based on the FE method simplifies the highly complex spinal system to a large extent, and factors such as the fibbers in the discs, muscle, and cyclic load are not considered. Third, due to the limitations of the available experimental data, only the intact model was validated, and the surgical models were developed using the intact model. In general, the biomechanical data obtained in the present study should be viewed as comparative data between different surgical cases due to the limitations of the model itself.

## 5 Conclusion

The PEEK rod internal fixation system may have better biomechanical properties than the titanium rod internal fixation system in delaying ASD, improving lumbar function in postoperative patients, and reducing the risk of screw loosening and breakage in lumbar long-segment instrumentation. Further studies are warranted to comprehensively validate the clinical outcomes for use of PEEK rods in lumbar long-segment instrumentation surgery in clinical practice.

## Data Availability

The raw data supporting the conclusions of this article will be made available by the authors, without undue reservation.
